# The Diagnostic–Therapeutic Care Pathway in Psoriasis: Towards ISO 9001:2015 Certification

**DOI:** 10.3390/medicina56050253

**Published:** 2020-05-22

**Authors:** Federica Veronese, Francesca Graziola, Edoardo Cammarata, Marco Andreassi, Vanessa Mazzoletti, Camilla Taglietti, Gaia Navarra, Paola Savoia, Rossana Tiberio

**Affiliations:** 1 AOU Maggiore della Carità, 28100 Novara, Italy; federica.veronese@med.uniupo.it (F.V.); francesca.graziola@maggioreosp.novara.it (F.G.); rossana.tiberio@med.uniupo.it (R.T.); 2 Department of Health Science, University of Eastern Piedmont, 28100 Novara, Italy; edoardocammarata@gmail.com (E.C.); ma.andreassi@gmail.com (M.A.); vanessa.mazzoletti@gmail.com (V.M.); 3 OPT, Consulenza di Direzione, 20159 Milano, Italy; camilla.taglietti@studioopt.com (C.T.); gaia.navarra@studioopt.com (G.N.)

**Keywords:** diagnostic–therapeutic care pathway, ISO 9001:2015 certification, quality management system, psoriasis, quality improvement

## Abstract

*Background and objectives:* Psoriasis (Pso) is a common skin condition characterized by a strong psychosocial impact, and is nowadays accepted as a systemic immune-mediated inflammatory disease. Diagnostic–Therapeutic Care Pathways (DTCPs) represent a predefined sequence of diagnostic, therapeutic, and assistance activities that integrate the participation of several specialists to obtain, for each patient, the correct diagnosis and thus the most appropriate therapy. A DTCP was validated in our dermatology clinic (AOU Maggiore della Carità, Novara, Italy). The validation process included the detailed elaboration of a protocol of diagnosis, staging of care, therapies, and follow-up of the patient with Pso. The formalization and adaptation of our DTCP resulted in ISO 9001: 2015 certification in May 2019. *Materials and methods:* This process involved several stages, including analysis of context and the identification of (i) targets, (ii) indicators, and (iii) service providers. The evaluation was based on a cohort of over 200 patients affected by moderate to severe Pso, who were treated and followed-up at our institution from September 2017 to April 2019. *Results:* The ISO 9001:2015 quality certification process allowed us to identify our weaknesses, i.e., the long waiting times for the first visit and the reduced physician–patient ratio, but also our strengths, such as the commitment to clinical research, effective collaboration with other specialists, the efficient use of technological and human resources, and attention to ensuring patient follow-up. *Conclusions:* In qualifying for and achieving the ISO Quality Management System (QMS) certification we were heartened to realize that our basic methodology and approach were fit for purpose. The implementation of the ISO QMS helped us to reorganize our priorities by placing the patient at the center of the process and raising awareness that Pso is not just a skin disease.

## 1. Introduction

Psoriasis (Pso) is a common skin condition characterized by a strong psychosocial impact. Nowadays it is accepted as a systemic immune-mediated inflammatory disease with complex pathogenesis and several comorbidities (i.e., psoriatic arthritis, mood disorders, cardiovascular disease, etc.) [1,2].

These aspects must be considered when the clinician addresses the needs of the psoriatic patient and develops the treatment plan; indeed, the therapeutic choice should be based on the individual patient, with the aim of targeting the condition precisely.

In November 2004, the Italian regulatory Activity of Pharmaceuticals Agency (AIFA) instituted the “Psocare” project, offering qualified healthcare personnel and ensuring effective, personalized, and cutting-edge therapies for Pso based on the latest best practices [3]. However, in Italy, there are enormous differences in patient care among the various regions, and validated Diagnostic–Therapeutic Care Pathways (DTCPs) are still poorly used [4].

The DTCP is a predefined sequence of diagnostic, therapeutic, and assistance activities that integrates the participation of several specialists to obtain, for each Pso patient, the correct diagnosis and thus the most appropriate therapy, taking into account the different comorbidities [5]. The aims of the DTCP are: (i) improvement of the care processes, (ii) identification of inefficiencies (cost, resources, etc.), (iii) expedited access to treatment, and (iv) an integrated multidisciplinary approach to the disease. The DTCP is based on tight clinical control, a precise definition of target treatment (treat-to-target), and a well-defined follow-up phase; it places the patient at the center of the process to create so-called “patient engagement”, i.e., a pact of alliance between doctor and patient [6].

**Figure 1:**
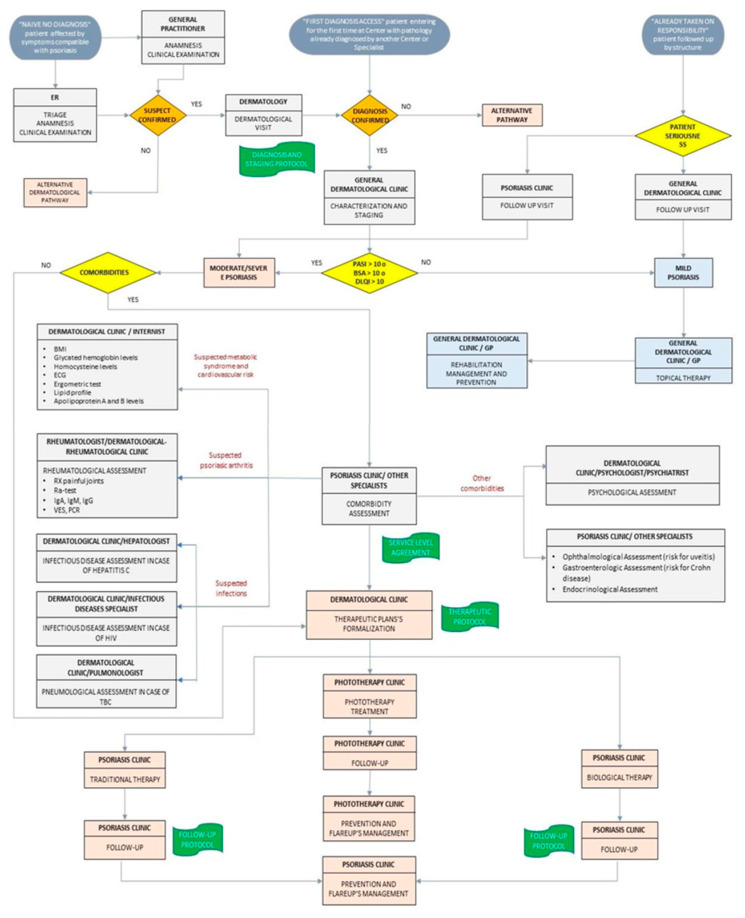
The validated Diagnostic–Therapeutic Care Pathway (DTCP) for Pso patients.

A DTCP was validated in our dermatology clinic (AOU Maggiore della Carità, Novara, Italy) (Figure 1). The validation process included a detailed elaboration of a protocol of diagnosis, staging of care, therapies, and follow-up of the patient with Pso. The formalization and adaptation of our DTCP resulted in ISO 9001: 2015 certification in May 2019.

### ISO 9001:2015

The international standard for Quality Management Systems (QMS) is based on legislation ratified by the UNI EN ISO 9000. This is a part of the ISO 9000 family of standards, i.e., quality specifications for all specific processes (for example environmental, occupational health and safety, food safety, and information and technology management processes, etc.) [7]. ISO 9001:2015 is the most recent version of ISO 9001 and applies to all organizations (institutions and health centers—public or private), regardless of size and geographical location, whether they be service providers, laboratories, or entire hospitals.

The concept of quality in health care is more difficult to define. According to the World Health Organization (WHO), the goal is to guarantee that the patient has access to the most appropriate diagnostic and therapeutic services in order to ensure the best health care [7].

**Figure 2:**
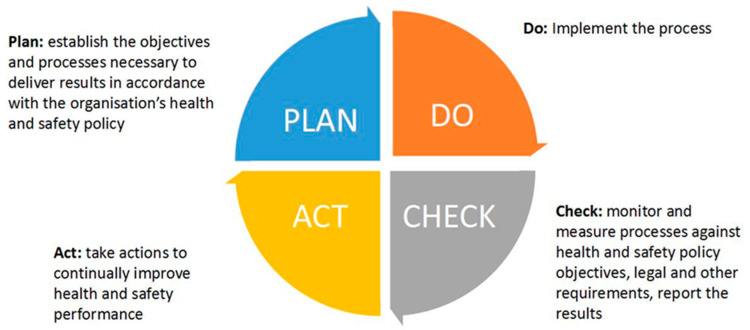
Deming cycle: Plan, Do, Check, Act (PDCA).

An effective QMS for a healthcare provider integrates the cardinal principles of ISO 9001 2015 with national and regional legislative requirements (accreditation systems), clinical indications of the scientific societies (good clinical practice), and programs of professional accreditation and quality improvement. QMS are based on the Deming cycle (Plan, Do, Check, Act—PDCA) in order to achieve continuous improvement [7,8] (Figure 2).

Certification of a health center improves its credibility and leads to an increase in users’ and stakeholders’ trust (patient’s family, general practitioners, region, state) by assuring the ability to meet needs and expectations.

The certification process consists of three phases, as shown in Table 1. If the outcome of the rating phases is appropriate, ISO 9001:2015 certification is awarded. In our specific case, the certifying entity was Bureau Veritas. 

## 2. Materials and Methods

Interventional studies involving animals or humans as well as other studies require ethical approval and must list the authority that provided approval and the corresponding ethical approval code. In 2017 we created a DTCP and a QMS, according to the criteria of UNI EN ISO 9001:2015, for patients affected by Pso at the dermatology clinic (AOU Maggiore della Carità, Novara, Italy), with the intention of qualifying for certification.

This process required several stages, including analysis of context and the identification of (i) targets, (ii) indicators, and (iii) service providers, as well as both internal and external audit procedures. The evaluation was based on a cohort of over 200 patients affected by moderate to severe Pso, some with psoriatic arthritis, who were treated and followed-up at our institution from September 2017 to April 2019. All subjects gave their informed consent to the treatment. At our Institution, the privacy policy is in accordance with the EU Regulation 2016/279 (Internal Regulation Number 1245, 30 December 2019).

## 3. Results

### 3.1. QMS and the ISO 9001:2015 Certification Process in Psoriasis Care 

#### 3.1.1. Analysis of Context

**Figure 3:**
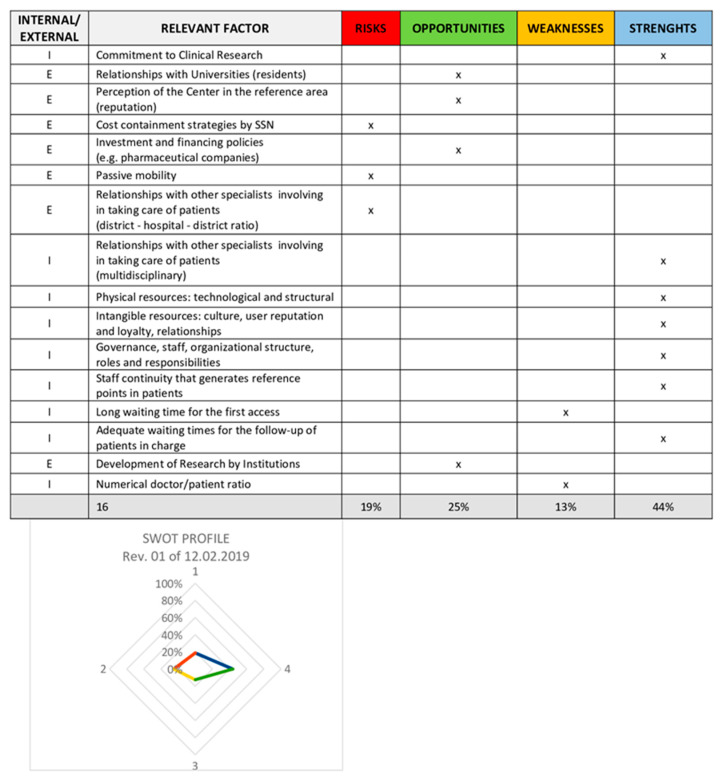
SWOT analysis.

We conducted a SWOT analysis (Strengths, Weaknesses, Opportunities and Threats; a strategic planning technique) to identify our internal strengths and weaknesses as well as external opportunities and threats. In this document, we detailed the different areas and activities of Pso care and defined the purpose and mission of the activity to be certified (i.e., the DTCP for patients affected by Pso and clinical research in our facilities) (Figure 3). In this phase, we produced a document explaining our quality policy and evaluated the satisfaction of both patients and staff through the use of questionnaires. The majority of patients gave a high score for the services provided by our center. Furthermore, most of our staff thought that their work was stimulating, recognized, and valued.

#### 3.1.2. Target Identification

From previous analyses and documents we established our targets, indicators, and actions to sustain them (Table 2). The education plan for years 2019–2020, including staff training events, was regarded as fit for purpose.

#### 3.1.3. Key Performance Indicator (KPIs)

The KPIs focused on specific areas such as structure (i.e., in our case indicators of activity, diagnosis, and appropriateness), processes, and outcomes. We identified 10 structural indicators, 2 outcome indicators, and 2 process indicators that are shown in Table 3.

#### 3.1.4. Service Provider’s Identification

Owing to the multiple comorbidities associated with Pso, the patient faces a multidisciplinary pathway that involves several specialists who are considered our service providers. We conducted a careful evaluation of the rheumatology, gastroenterology, pulmonology, hepatology, diabetology, hospital pharmacy, and phototherapy services to identify critical issues and to better standardize our processes.

#### 3.1.5. Internal Audit

Once the information was collected and the documentation produced, we performed an internal audit to identify any weaknesses and failures. In all documents, date and version numbers were included. This process detected some non-conformities that were corrected before the external audit took place. Based on the lessons learned we developed an improvement plan (Table 4).

#### 3.1.6. External Audit

**Figure 4:**
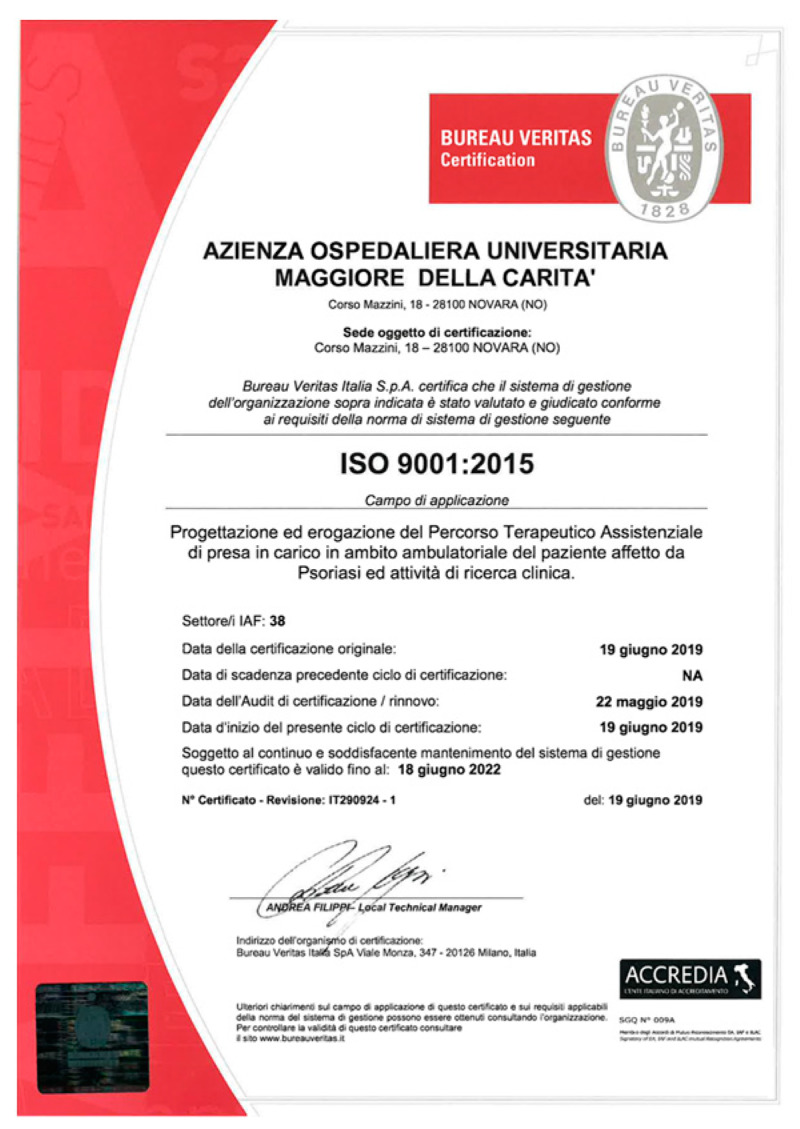
ISO 9001:2015 certificate.

During the external audit (May 2019) the documentation was checked and the auditor reviewed and compared all documents produced; moreover, different members of staff were interviewed to evaluate their awareness of the certification’s process. The auditor gave us a positive and favorable report on 19 June 2019 (Figure 4). 

The ISO 9001:2015 certification was awarded for the design and delivery of the DTCP for patients affected by Pso and clinical research in our dermatology clinic.

#### 3.1.7. Annual Revision

In order to maintain our certification, procedures are reviewed annually and the certificate renewal procedure occurs every 3 years.

### 3.2. Real-Life Experience in QMS and ISO 9001:2015 Certification Process

Data analysis conducted in our center during the QMS implementation and the ISO 9001:2015 certification processes showed that:

1. For about 10% of Pso patients, the waiting time between the first diagnosis and referral to the dermatologist was of several years. This clearly outlines a need for better patient education regarding the characteristics and the treatment possibilities of this pathology. Toward this end, we aim to draft information brochures on Pso and its comorbidity to be widely distributed to patients [9].
2. In Italy, about 45% of Pso patients are referred to a specialist hospital by their general practitioner (GP) [9]. However, in our cohort, the majority of Pso patients (62.5%) were referred by other hospital specialists. Therefore, we intend to organize a working group with GPs to facilitate patient referral and early diagnosis.
3. In our sample, the average Psoriasis Area Severity Index (PASI) at diagnosis was between 5 and 9, but in the literature this was between 5 and 7.4 [10]. We assume that this variance is due to the delay in referring patients.
4. In our sample, psoriatic arthritis represents the most frequently observed comorbidity, followed by inflammatory bowel disease (IBD) and cardiovascular disorders according to the literature [11,12,13,14]. For this reason, we drew up a “Service Level Agreement”, a document in which we identified, for each comorbidity, the referring specialist, the reasons for the visit, and the elapsed time between referral and the visit. In the future, we plan to organize meetings or collegial visits for the management of more complex conditions.
5. We switched therapies in about 31% of patients. This indicates our confidence in assessing the severity of the disease from the beginning and in administering the most suitable therapy for the patient, ensuring that our approach is based on the most recent guidelines.
6. In our experience, all patients treated with biological medicines maintained the pre-established follow-up intervals of between 3 or 6 months. This demonstrates the patient’s awareness of the importance of the therapy and indicates a high level of satisfaction in our services. Further, this validates the efficiency of communication between specialists and patients.
7. However, in our center the waiting time for the first visit was still long (about 100 days). We have planned strategies to reduce the waiting time, mainly through the acquisition of new resources.

The ISO 9001:2015 quality certification process allowed us to identify our weaknesses, i.e., the long waiting times for the first visit and the reduced physician–patient ratio, but also our strengths: the commitment to clinical research (publications in indexed journals and participation in conferences as speakers or auditors), effective collaboration with other specialists, efficient use of technological and human resources, and attention to ensuring patient follow-up.

## 4. Discussion

Nowadays, accountability, good management, and continuous improvement are essential requirements for good service. The benefits of the implementation of an ISO 9001:2015 QMS are the systematization and standardization of the processes, the continuous updating of the documentation, the optimization of resources, and the timely detection of failures. Specifically, the correction of irregularities and adverse events detected during the audits is a useful implementation tool.

This process leads to a higher level of satisfaction for both the patients and other stakeholders (i.e., healthcare management professionals), and compliance of the services offered with the established quality requirements accredited through an external body [7].

Moreover, the ISO certification confirms that the treatments offered as well as research and teaching correspond to quality standards and guarantee compliance for clinical trials.

In public health, the process of certification is commonly applied to centralized services such as analysis laboratories [7], while in clinical services it is not fundamental to demonstrate compliance with quality standards. To the best of our knowledge, in Italy our dermatology clinic was the first to obtain this recognition for Pso care.

For clinicians, completing this path was difficult because it was necessary to become confident with the nomenclature used in the ISO QMS. Moreover, a high level of self-criticism is needed to detect when the pre-established standards are not met and implement remedial actions. To these ends, it was necessary to record, test, and hone for several months throughout the internal audit in order to prepare for certification. The costs of acquiring certification (which often require the acquisition of a dedicated grant) and the difficulties in obtaining funding in the public sector must also be considered.

## 5. Conclusions

In qualifying for and achieving ISO QMS certification we were heartened to realize that our basic methodology and approach were fit for purpose. The implementation of the ISO QMS helped us to reorganize our priorities by placing the patient at the center of the process and raising awareness that Pso is not just a skin disease.

We believe that by putting patients “at the center” increases patient satisfaction and most importantly improves treatment outcomes, reduces concerns, and improves compliance with best practice. Having a QMS means being able to ensure continuity of patient care and access to specialist care, pharmacological options, and more effective treatments; moreover, it means the chance to participate in collaborative research programs, share recommendations for the management of the patient with Pso, and, not least, to optimize costs.

We hope this approach promotes the integration among hospital specialists and between specialist centers and the outside world, particularly with general practitioners in order to achieve economies of scale and efficiencies in controlling healthcare costs whilst offering the patient an organized process and simplified clinical assistance.

## Figures and Tables

**Table 1:** The three macro-processes of certification.

Preliminary analysis of context and interested parties Accurate risk and opportunity analysis following risk-based thinking Identification of aims and definition of managerial, clinical, and result indicators to achieve them through the quality policy and mission	Definition of guidelines and technical, organizational, and structural standards of the final result Personalization and humanization of services Improvement of waiting times Monitoring of patient/user and hospital staff satisfaction	Identification of adverse events (unconformity) and implementation of corrective and preventing actions to improve overall quality management Constant measurement, through analysis and data collection, of the quality of services/performance Evaluation of service providers (e.g., other hospital units) to ameliorate them

**Table 2:** Targets, indicators, and action.

Targets	Indicators	Actions/Commitments	Responsible Party
Reduction of the waiting time from referral to first visit through an improvement of appropriateness in access to the psoriasis center	Time difference between referral and the first visit	Create a document for general practitioners and territorial specialists which serves as guideline for correct identification of psoriasis patients for the center	Working group
Improved treatment adherence	Proportion of patients lost to follow-up	Raise awareness of the importance of treatment adherence. Create a survey to evaluate patients’ comprehension.	Working group
Implementation of a dedicated nurse	Obtained dedicated nursing staff	Communicate to the medical director the need for dedicated nursing staff.	Medical director and hospital medical management
Increased resources for medical students and residents	Number of participants at national and international courses Number of publications	Involve students and residents in clinical and basic research activities.	Working group
Increased participation in clinical trials	Number of clinical trials	Identify barriers to clinical trial participation to enable interventions that could help to increase participation.	Working group

**Table 3:** Key performance indicators (KPIs).

Structural Indicators (Activity, Diagnosis and Appropriateness)	Outcome Indicators	Process Indicators
Number of patients Number of new patients Access channel to the center Total number of visits Disease severity (PASI-BSA-DLQI) Percentage of patients sent for multidisciplinary assessment Enhancement of multidisciplinary assessments Volume of treatments Compliance with the one-month follow-up for patients under systemic biological therapy Compliance with the three-month follow-up for patients under systemic biological therapy	Percentage of patients achieving PASI 75 Percentage of patients achieving PASI 90	Waiting time for first visit Waiting time for biological treatment

**Table 4:** Plan of improvement.

Action	Times	Responsible Party
Attracting the interest of staff regarding report policy disclosure in accordance with company policy	July 2019	Medical director
Attracting interest of staff regarding ministerial recommendations through periodic meetings	December 2019	Medical director
Sharing, with all the staff, performance indicators of the psoriasis center	December 2019	Medical director and working group
Sharing, with colleagues inside the hospital and with those in the district, the certification obtained by the center	December 2019	Medical director and working group
